# Feasibility of Data Collection Via Consumer-Grade Wearable Devices in Adolescent Student Athletes: Prospective Longitudinal Cohort Study

**DOI:** 10.2196/54630

**Published:** 2025-06-13

**Authors:** Danielle Ransom, Brant Tudor, Sarah Irani, Mohamed Rehman, Stacy Suskauer, P Patrick Mularoni, Luis Ahumada

**Affiliations:** 1 Johns Hopkins All Children’s Hospital, 880 Sixth Street South, Suite 460, St. Petersburg, FL, 33701, United States, 1 7277674824; 2 Johns Hopkins University School of Medicine, Baltimore, MD, United States; 3 Kennedy Krieger Institute, Baltimore, United States

**Keywords:** sports-related injuries, wearable technology, recovery monitoring, adolescent student athletes, concussion, orthopedic injuries, orthopedic, musculoskeletal issues, sport, sports, injury, injuries, athlete, athletes, athletics, recover, monitor, monitoring, students, sensors, Fitbit, data collection, wearable, wearables, adolescent, adolescents, teen, teens, teenager, teenagers

## Abstract

**Background:**

Recent advancements in sports medicine have been fueled by innovative technologies, particularly consumer-grade wearable devices like Fitbit, Apple Watch, and Garmin. These devices offer physiological and biomechanical data and hold promise for personalized, real-time, and remote assessment of athlete recovery. However, few studies have been conducted with these devices in adolescent student athletes.

**Objective:**

The primary objective of this study was to assess the feasibility of integrating consumer-grade wearable technology into injury recovery monitoring of adolescent student athletes.

**Methods:**

The study included 34 high school student athletes aged 14‐18 diagnosed with either concussion or orthopedic injury, enrolled within 10 days of injury. Participants were equipped with a Fitbit Sense for continuous monitoring of physiological markers, including cardiovascular metrics, physical activity levels, and sleep patterns. Data collection extended 4‐6 weeks beyond injury clearance, during which adherence rates were assessed at both hourly and daily intervals. Hourly adherence was defined as the proportion of participants with at least 1 recorded heart rate data point per hour, while daily adherence was defined as the proportion of participants with at least 1 recorded heart rate data point per 24-hour period.

**Results:**

The study demonstrated high participant adherence to wearing the device. The orthopedic injury cohort exhibited a median adherence rate of 95%, with individual rates ranging from 82% to 100%. Similarly, the concussion cohort demonstrated a median adherence rate of 93%, with adherence rates spanning from 37% to 100%. Notably, the study encountered minimal issues related to device functionality, with only 1 participant necessitating a device replacement.

**Conclusions:**

These findings demonstrate successful integration of wearable technology in data collection for adolescent student athletes recovering from sports-related injuries. However, it is important to consider current limitations, including factors that may influence data accuracy and precision. In conclusion, this feasibility study demonstrates the practicality of using consumer-grade wearable technology for the collection of physiological and biomechanical parameters in adolescent student athletes recovering from sport-related injuries. The high level of adherence highlights the potential applicability of consumer-grade wearable devices in this population. Study findings lay the foundation for future investigations with larger and more diverse cohorts to identify the utility of device metrics in identifying unique patterns of injury-specific recovery (ie, sport-related concussion). Consumer-grade wearable devices offer promise for optimizing assessment and management of injured athletes through wearable technology integration into standard clinical protocols.

## Introduction

Common sport-related injuries, including concussion and orthopedic trauma, have the potential to present significant challenges to athletes beyond restricted participation in sports [1-3]. Concussion, in particular, is a growing concern due to the acute impact of recovery on activities of daily living [4-6]. Orthopedic injuries, on the other hand, can disrupt an athlete’s training regimen and potentially lead to long-term musculoskeletal issues if not properly managed. Prolonged recovery from injuries of any type has the potential to contribute to heightened symptoms of depression and anxiety compared with uninjured athletes [2,7].

Complications during recovery from concussion and orthopedic injuries are often minimized with appropriate intervention and management [5,7]. However, disparities exist in access to evidence-based care, with up to 30% of surveyed individuals in the United States reporting that they did not obtain health care for a sports-related injury [8]. Unlike collegiate and professional athletes who are often provided with acute care within a network of affiliated health care specialists, access to optimal care pathways is less clear for adolescent student athletes following sport-related injury [9,10]. Youth rely on the availability of parents or other caregivers to identify, coordinate, and provide transportation to specialists for injury monitoring and rehabilitation while co-managing their academic load and meeting the expectation for full-time school attendance [11]. Student athletes who are unable to access care or adhere to follow-up rehabilitation following sport-related injury are at higher risk for prolonged recovery and residual injury effects on physical and mental health [2,7,12]. Given these challenges, it is imperative to investigate novel, minimally invasive, clinically viable, and readily accessible approaches—such as remote monitoring and data collection in the child’s typical environment—to effectively support adolescent student athlete injury recovery.

The field of sports medicine has made notable advancements in recent years through innovative technologies with the potential to improve the assessment and management of injured athletes [13,14]. Among these technologies, consumer-grade wearable devices such as Fitbit, Apple Watch, and Garmin have garnered considerable attention for their ability to provide continuous, real-time physiological and biomechanical data in order to provide comprehensive monitoring of individual athletes’ health and performance [15,16]. The integration of these devices with standard clinical protocols offers a promising solution to the challenges of recovery monitoring [17-20]. Consumer-grade wearable devices provide a noninvasive and user-friendly means to collect a multitude of physiological and biomechanical parameters, including heart rate, activity levels, sleep patterns, and movement patterns [18]. Data gleaned through these devices could enable health care providers, coaches, and athletes themselves to make informed decisions regarding training intensity, physiologic adaptation, and overall well-being [21]. However, integrating wearable technology in health care research is not without challenges. Issues such as data accuracy, participant adherence, ethical considerations, and the interpretability of data must be carefully navigated [17,18,21]. Moreover, the diverse nature of injuries and individual variations among athletes necessitate a nuanced approach that considers the unique presentation of each patient [22].

This feasibility study aimed to assess the practicality of integrating consumer-grade wearable technology into the recovery monitoring of adolescent student athletes through an evaluation of device usability and participant adherence across commonly measured physiological and biomechanical parameters, including cardiovascular metrics, physical activity levels, and sleep patterns.

## Methods

### Participants

The study sample is comprised of 2 age- and sex-matched cohorts of high school student athletes aged 14‐18 years who were actively participating in their sports season at the time of enrollment (September 2021-June 2022). One cohort included male and female athletes participating in sports who were diagnosed with concussion, as defined by the 2016 Berlin International Consensus Conference for Concussion in Sport [23]. The second cohort consisted of athletes diagnosed with an acute, nonsurgical orthopedic injury. Eligible participants were evaluated and diagnosed with a concussion or acute, nonsurgical orthopedic injury within 10 days of their injury in an ambulatory sports medicine clinic; had access to a reliable internet connection and cell phone or home computer; and were able to read, understand, and comply with study instructions. Participants were additionally required to attend 2 separate study neurocognitive screening visits for study inclusion.

Participants were excluded from the study if they met any of the following criteria: prior traumatic brain injury within the past 6 months; history of moderate to severe traumatic brain injury (Glasgow Coma Scale<13) at any time; brain mass, prior neurosurgery, or central nervous system disorder; moderate to severe cognitive dysfunction or structural brain disease or malformation; or participation in more than 1 sport during the time of enrollment. Additionally, if a participant had not yet recovered from their injury, the study team discontinued protocol participation 180 days post enrollment.

### Ethical Considerations

This study was approved by the Johns Hopkins All Children’s Hospital institutional review board (IRB00457018 for data collection; IRB00211758 for data analysis and reporting). Informed consent was obtained from participants and their legally authorized representatives, and participation was voluntary. To ensure privacy and confidentiality, data were deidentified and transmitted securely to the Fitbit cloud-based database via a HIPAA (Health Insurance Portability and Accountability Act)-compliant protocol, allowing access to deidentified data through a code link on the Fitabase platform. Participants received compensation, including a Fitbit Sense, which they kept after study participation ended; two US $50 gift cards distributed at enrollment and upon completion of the study protocol; and 6 volunteer hours that could be applied toward high school service requirements.

### Wearable Device

The Fitbit Sense (Fitbit Inc) is a wrist-worn wearable device designed for continuous monitoring of various physiological parameters, such as heart rate, resting heart rate, step counts, daily minutes of vigorous activity, and sleep patterns and architecture. This wearable device was chosen because of its long battery life (up to 6 d) and its durability, including waterproofing. The Fitbit Sense is equipped with a range of sensors, including a photoplethysmogram sensor, an optical heart rate sensor, an electrodermal activity sensor, an accelerometer, a gyroscope, and an ambient light sensor. The photoplethysmogram sensor uses green and red light-emitting diodes along with a photodiode to measure blood volume changes in the microvasculature of the skin, allowing for the estimation of heart rate and blood oxygen saturation. The optical heart rate sensor uses a combination of green and infrared light-emitting diodes to capture heart rate data through the measurement of blood flow variations. To detect motion, the accelerometer and gyroscope work in tandem to monitor physical activity and provide information on movement patterns. The accelerometer quantifies linear motion, enabling accurate tracking of steps, distance, and intensity of physical activity. The intensity and duration of these activities are recorded and used to estimate metrics like energy expenditure, active minutes, and overall physical activity levels. The gyroscope measures angular velocity, facilitating the detection of activities like cycling, swimming, and rotational movements. To detect sleep, the Fitbit Sense uses a combination of the accelerometer and heart rate data to discern different sleep stages, including sleep onset and wakefulness, as well as light, deep, and rapid eye movement sleep.

### Data Collection

After obtaining written consent from participants and their legally authorized representatives, the study research assistant provided each participant with a Fitbit Sense, along with a device tutorial covering full-time wear, daily syncing with the Fitbit application, and standardized charging times (twice weekly during evening downtime at home). The study team assisted participants with downloading the Fitbit smartphone application, creating an account linked to a study-generated deidentified email address managed by the team, and connecting their provided Fitbit Sense to a smartphone or compatible computer via Bluetooth. To prevent the collection of identifiable protected health information, the study team disabled GPS functionality by default and instructed participants not to alter this setting on their device or the smartphone app for the duration of the study. Once the Fitbit Sense was connected, participants were asked to wear the device for as much of their day as possible, including sleep. Patient-generated data were then transmitted to the Fitbit cloud-based database through the internet-connected application. Fitbit then connected to the data management platform, Fitabase (Small Steps Labs LLC), via HIPAA-compliant protocol to allow investigators to access the deidentified data via code link. The study team monitored the frequency of application syncing and contacted participants via phone or SMS text message to provide troubleshooting if devices were synced less than twice weekly. Data points were gathered across 3 key parameters: cardiovascular, physical activity, and sleep. Cardiovascular data included heart rate (beats/minute) and resting heart rate. Physical activity data included daily step count and daily minutes of physical activity, categorized into vigorous, moderate, light, and sedentary exertion levels. Sleep data included total daily minutes spent asleep, minutes of nighttime wakefulness, and sleep architecture characterization based on time spent per night spent in deep, light, wakeful, and rapid eye movement states.

As part of standard clinical care, participants were monitored by a sports medicine physician in clinic every 1‐2 weeks until clearance criteria were met. Criteria for concussion clearance included clinical examination by a specialized health care provider to confirm resolution of acute symptoms, return to preinjury symptom levels during rest and activity, tolerance for cognitive and physical exertion, and successful completion of a supervised gradual return-to-play protocol. Length of concussion recovery was defined as the number of days between the date of injury and the date the participant received medical clearance to begin a gradual return-to-play protocol based upon symptom resolution at rest and return-to-learn [24]. Criteria for orthopedic clearance included clinical examination by a sports medicine physician to confirm injury stabilization, satisfactory range of motion and strength, appropriate weight-bearing status (if applicable), demonstrated ability to perform functional activities without significant impairment, well-managed pain levels, absence of indications for immediate surgery, and medical clearance confirming fitness for sport participation. Length of orthopedic recovery was defined as the number of days between the date of injury and the date the participant received medical clearance to participate in sport. Participant adherence was continuously monitored from enrollment to approximately 4‐6 weeks after meeting injury clearance criteria. At that point, the study team initiated completion procedures, which included transferring the Fitbit account to the participant and discontinuing data tracking. If a participant had not yet recovered from their injury, protocol discontinuation was initiated by the study team 180 days post enrollment.

### Statistical Analysis

Statistical analyses were performed using the *Pandas* and *NumPy* Python packages (Python Software Foundation). Continuous variables were summarized with means, SDs, quantiles, and ranges (minimum to maximum). Categorical variables were summarized with counts and percentages. Adherence to device use, the primary feasibility outcome, was assessed through both hourly and daily granularity, based on the presence of recorded heart rate data. *Hourly adherence* was defined as the proportion of participants with at least 1 recorded heart rate data point per hour. If heart rate data were collected during a given hour, a subject was deemed to have complied for that hour. Presented here are the averages for said hour over every day in each 2-week period from enrollment until study completion. For example, with 336 hours in a 2-week period, an overall adherence rate of 1 would indicate that all actively participating subjects in the cohort wore their (functioning) device for at least a portion of each of the 336 hours that comprised that 2-week period. Here, day was defined as 7 AM to 6:59 PM while night was defined as 7 PM to 6:59 AM according to each participant’s local time zone. *Daily adherence* was defined as the proportion of participants with at least 1 recorded heart rate data point per 24-hour period. Patterns of data capture revealed high initial adherence, with missed data points increasing over time. Extending the previous example, a daily adherence rate of 1 for a given 2-week period would indicate that the subject’s Fitbit recorded at least 1 heart rate data point during each of the 14 days occurring in the period. If a study participant completed the study during a given 2-week period, a daily adherence rate of 1 would indicate that the subject’s Fitbit recorded at least 1 heart rate during each of the days in which they were actively enrolled in the study (eg, 5/5 d). To evaluate feasibility over time, adherence metrics were analyzed across study participation intervals (ie, days [1-28] and [29-41]).

## Results

### Sample Characteristics

A total of 38 participants were enrolled in the study between September 2021 and April 2022. In total, 4 participants were excluded from analyses due to nonadherence with study procedures, including failure to attend the required second study visit (n=2, orthopedic injury cohort), failure to wear the Fitbit device (n=1, orthopedic injury cohort), and lack of medical clearance by 180 days post enrollment (n=1, concussion cohort). The final study sample consisted of 34 participants (n=17 concussion cohort; n=17, orthopedic injury cohort).

Participants were evenly distributed by sex (female: 61.8%) and matched by age, with a mean age of 15.71 (SD 1.15) years. Participants engaged in a variety of sports, with 71% of the concussion cohort and 58% of the orthopedic injury cohort participating in contact and collision sports. The concussion cohort had a higher proportion of student athletes participating in contact and collision sports (71%) compared with the orthopedic injury cohort (58%). The majority of participants were White and non-Hispanic. Demographic characteristics are presented in Table 1.

**Table 1.** Demographic and injury characteristics of adolescent student athlete cohorts enrolled in this prospective longitudinal study.

	Concussion (n=17)	Orthopedic injury (n=17)	Overall (N=34)
Age (years), mean (SD)	15.76 (1.11)	15.65 (1.19)	15.71 (1.15)
Gender, n (%)			
Male	7 (41.2)	6 (35.3)	13 (38.2)
Female	10 (58.8)	11 (64.7)	21 (61.8)
School grade, n (%)			
9	6 (33.3)	5 (29.4)	11 (32.4)
10	3 (16.7)	6 (35.3)	9 (26.5)
11	5 (27.8)	5 (29.4)	10 (29.4)
12	3 (16.7)	1 (5.9)	4 (11.8)
Race, n (%)			
White	14 (82.4)	9 (52.9)	23 (67.6)
Black	1 (5.9)	6 (35.3)	7 (20.6)
Other	2 (11.8)	2 (11.8)	4 (11.8)
Ethnicity, n (%)			
Hispanic	2 (11.8)	5 (29.4)	7 (20.6)
Non-Hispanic	15 (88.2)	12 (70.6)	27 (79.4)
Sport of injury, n (%)			
Soccer	5 (29.4)	6 (35.3)	11 (32.4)
Football	5 (29.4)	1 (5.9)	6 (17.6)
Basketball	1 (5.9)	2 (11.8)	3 (8.8)
Softball	2 (11.8)	1 (5.9)	3 (8.8)
Track	0 (0)	3 (17.6)	3 (8.8)
Volleyball	2 (11.8)	1 (5.9)	3 (8.8)
Baseball	0 (0)	2 (11.8)	2 (5.9)
Cheerleading	1 (5.9)	0 (0)	1 (2.9)
Flag football	0 (0)	1 (5.9)	1 (2.9)
Rugby	1 (5.9)	0 (0)	1 (2.9)
Recovery (days), mean (SD)	25.64 (18.1)	32.12 (20.48)	--

### Adherence to Device Use

#### Overall Trends

**Figure 1.**
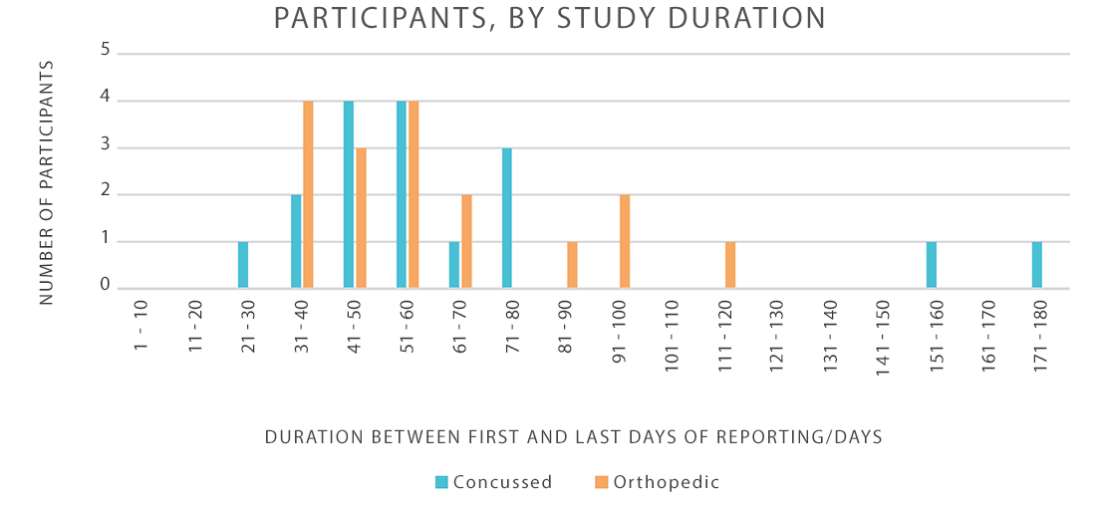
Duration of study participation and daily device adherence in concussion and orthopedic injury cohorts.

Adherence to device wear was examined at both hourly and daily intervals across the study period. Median daily adherence rates were high across cohorts, with a 93% (range 37%‐100%) adherence rate in the concussion cohort and a 95% (range 82%‐100%) adherence rate in the orthopedic injury cohort. The average study duration was 66 days for the concussion cohort and 61 days for the orthopedic injury cohort. The majority of participants (67.6%) actively contributed data for 33‐61 days (Table 2 and Figure 1). No significant issues with device connectivity or functionality were reported during the study period; however, 1 participant lost the device and required a replacement.

**Table 2.** Daily adherence rates to wearable device monitoring by study interval across concussion and orthopedic injury cohorts.

Patient number	Study duration (days)	Totaldays reporting	Overall dailyadherence rate	Daily adherence rate			
				Days1‐14	Days15‐28	Days29‐42	Days43‐56
Concussion cohort							
1	52	35	0.67	0.93	0.71	0.36	0.70
2	59	22	0.37	0.93	0.57	0.00	0.00
3	75	75	1	1	1	1	1
4	28	24	0.86	1	0.71	—	—
5	33	28	0.85	0.93	0.71	1	—
6	39	30	0.77	0.86	0.93	0.45	—
7	72	61	0.85	1	0.71	1	1
8	42	42	1	1	1	1	—
9	56	56	1	1	1	1	1
10	154	149	0.97	1	1	1	1
11	74	52	0.70	1	0.36	0.43	0.79
12	49	47	0.96	1	1	0.86	1
13	180	155	0.86	1	0.93	0.79	1
14	43	40	0.93	0.93	0.86	1	1
15	61	59	0.97	1	0.93	1	0.93
16	49	47	0.96	1	0.93	0.93	1
17	56	54	0.96	1	1	0.86	1
Overall	66.0	57.4	0.86	0.98	0.84	0.79	0.88
Orthopedic injury cohort							
1	35	34	0.97	1	1	0.86	—
2	99	82	0.83	1	1	1	0.00
3	59	56	0.95	1	1	1	0.79
4	53	52	0.98	1	1	1	0.91
5	50	43	0.86	1	1	0.50	1
6	95	79	0.83	1	1	1	1
7	47	47	1	1	1	1	1
8	49	48	0.98	1	1	0.93	1
9	118	114	0.97	1	1	1	1
10	56	46	0.82	1	1	0.71	0.57
11	40	39	0.97	1	1	0.92	—
12	40	37	0.93	1	0.86	0.92	—
13	61	57	0.93	1	1	1	0.79
14	81	79	0.98	1	1	0.93	0.93
15	64	56	0.88	1	1	0.79	0.86
16	54	50	0.93	0.93	1	0.86	0.92
17	33	33	1	1	1	1	—
Overall	60.8	56.0	0.93	1.00	0.99	0.91	0.83

#### Adherence Over Time

Daily adherence remained high across time intervals, though some variability was observed as participants completed the study at different time points based on individual recovery timelines. Adherence was highest in the initial 2 weeks, with median rates of 98% in the concussion cohort and 100% in the orthopedic injury cohort (Table 2). During the first 2 weeks of participation (days 1‐14), nearly all participants maintained near-perfect adherence, with rates of 98% in the concussion cohort and 100% in the orthopedic injury cohort. In the following 2-week period (days 15‐28), adherence remained high (84% concussion, 99% orthopedic injury), though a slight decline was observed as some participants completed the study upon meeting clearance criteria and were no longer enrolled. By days 29‐42, adherence rates showed a moderate decrease (79% concussion, 91% orthopedic injury), again reflecting study completion rather than disengagement. In the final observed time interval (days 43‐56), fewer participants remained enrolled due to individual recovery timelines, but those still actively participating sustained strong adherence, with rates of 88% in the concussion cohort and 83% in the orthopedic injury cohort. Rather than disengaging, participants who met clearance criteria exited the study, leading to expected decreases in the number of active participants over time.

#### Day Versus Night Adherence

**Figure 2.**
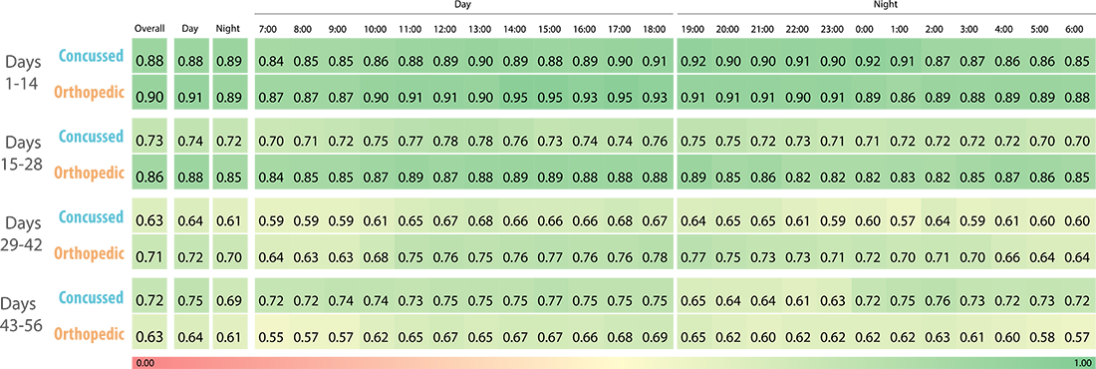
Hourly adherence rates by time of day (day vs night) and injury cohorts.

Adherence rates were comparable between daytime and nighttime periods (Figure 2), suggesting that participants were comfortable wearing the device overnight and willing to sustain compliance outside of structured daily activities. No significant differences were observed between cohorts.

#### Data Capture and Physiological Monitoring

**Figure 3.**
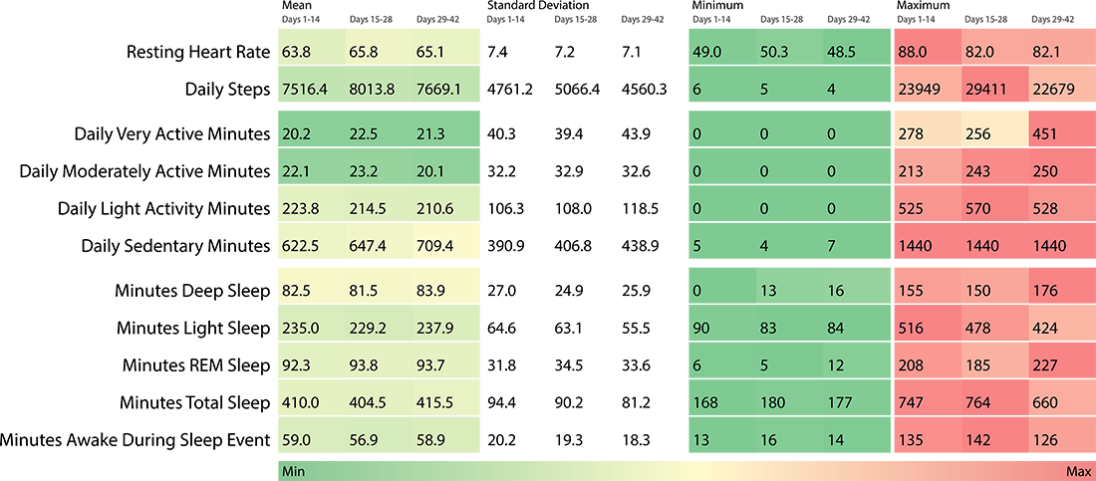
Heat map of physiological parameters collected longitudinally via Fitbit Sense during injury recovery. REM: rapid eye movement.

Participant-generated data provided continuous, real-world monitoring of cardiovascular metrics, physical activity levels, and sleep patterns across the study period. Data completeness was highest in the early weeks, with gradual declines over time as participants completed the study upon recovery clearance. Physiological data collected across time intervals are summarized in Figure 3, demonstrating the range and variability of parameters measured.

## Discussion

### Principal Findings

This feasibility study demonstrated the practicality of integrating consumer-grade wearable technology for the collection of physiological and biomechanical parameters in a pilot sample of adolescent student athletes recovering from sport-related injuries. High adherence rates across both cohorts highlight the potential for real-world implementation of wearable devices in clinical and research settings. Factors such as long battery life and ease of use likely contributed to sustained adherence, while the active engagement of student athletes in their sport season may have further motivated participation [25-27].

As expected, daily adherence declined, and greater variability was observed between cohorts as the study progressed, highlighting important considerations for future longitudinal study designs. Specifically, the observed differences in daily adherence rates between the 2 cohorts suggest varying levels of participant adherence with the device in the latter two-thirds of the study. Of note, this time period may have coincided with a gradual return to sport participation. Student athletes with orthopedic injuries demonstrated consistently higher rates of daily adherence compared to those with concussion, indicating a greater willingness or ability to adhere to wearing the Fitbit. For example, most contact and collision sports limit arm and wrist accessories during practice and competition due to safety precautions. If a student athlete was required to remove their device for practice or game play, they may have been less likely to remember to put it back on than they would have for standard charging occurring at home. The slightly lower adherence rates observed for student athletes recovering from concussion may be attributed to the higher number of student athletes from contact and collision sports included in this cohort compared with the orthopedic injury cohort. This suggests that wearable technology may be more readily embraced by certain patient populations and may be influenced by factors such as perceived relevance to their recovery process, sport-specific rules and regulations, or motivational factors. This finding highlights the importance of considering individual student athlete characteristics and needs when implementing such devices in study protocols. Strategies to enhance participant adherence, such as sport-specific procedures for device usage upon return to play and real-time feedback from the research team after a set interval of nonadherence, may be necessary to optimize data capture in studies with adolescent student athletes. Additional domain-specific considerations are presented below.

### Cardiovascular Monitoring

Consumer-grade wearable devices offer promise for health care research through real-time monitoring of cardiovascular parameters. This feasibility study provides support for future investigations using these devices for cardiovascular monitoring following sport-related injuries. Variability in resting heart rate over time observed in these data may reflect the impact of limited activity acutely post injury, followed by a transient increase due to heightened sympathetic activity and subsequent adaptation associated with autonomic balance and cardiovascular efficiency as participants returned to sport [28,29]. However, it is important to acknowledge known limitations, including device placement, individual skin type, skin complexion, and user adherence, may influence the accuracy and reliability of the collected data [18]. Continuous heart rate monitoring may not capture transient physiological changes during intense, short-duration activities [30-32]. While these limitations must be considered when interpreting the data, access to continuous cardiovascular patterns provided by consumer-grade wearable devices remains a valuable tool for future study protocols aimed at tailoring rehabilitation interventions, adjusting exercise regimens, and assessing overall recovery progress [33].

### Physical Activity Monitoring

This study highlights the broad feasibility of using wearable technology to comprehensively monitor physical activity in adolescent student athletes. Physical activity data, including step counts and exertion levels, gathered from wearable devices have the potential to provide nuanced understanding of participants’ activity patterns, which is essential for fine-tuning rehabilitation strategies [34,35]. Tracking changes in step counts over time can serve as a valuable indicator of progress in regaining mobility and function post injury [36]. However, there are limitations consistent with those discussed within the cardiovascular parameters. The accuracy and precision of consumer-grade wearable devices may vary based on factors such as device calibration, placement, and individual or sport-specific characteristics, which can contribute to discrepancies in the recorded data [16]. In addition, wearable devices may not accurately capture certain activities, particularly those involving nonstandard movements or activities that do not primarily rely on step-based motion [37]. Although participants demonstrated high levels of broad adherence to device use during this study, the consistency in step counts and activity levels over time is unexpected, given that injured student athletes would presumably demonstrate an increase in exertion over time as they returned to sport. As noted earlier, sport-specific rules and regulations may have limited use during practice and game play, which may be reflected in the separate and incongruent finding of increasing sedentary minutes over time. Since physical activity metrics gleaned through the wearable device relied on cardiovascular exertion and step counts, nonuse of the device during primary sport participation could have limited data capture. Of note, resting heart rate is less likely to be impacted by this limitation since data are gathered at night. Monitoring activity levels using wearable devices may offer important insights into the rehabilitation process following sport-related injury but may be limited by adherence to sport-specific regulations during activity participation and should be considered by investigators when developing future study protocols using these devices.

### Sleep Monitoring

Sleep data capture using a consumer-grade wearable device may provide valuable insights into participants’ sleep patterns during injury recovery. Understanding the quality and duration of sleep is integral to assessing the restorative processes crucial for injury recovery, particularly for athletes recovering from concussion [38,39]. Findings from this study support the feasibility of collecting a range of sleep parameters that could impact injury recovery, including time spent asleep versus awake at night and specific sleep architecture patterns. However, it is imperative to acknowledge limitations associated with gathering sleep data via consumer-grade wearable devices. While this technology has made significant strides in sleep tracking, accuracy may still be influenced by factors such as device positioning, individual sleep habits, and variations in sleep stages [40]. Devices may not capture certain nuances of sleep architecture that are crucial for a comprehensive understanding of sleep quality [40]. Factors like sleep disorders or environmental disturbances may also introduce variability in the recorded data. It is important to note that while wearable-based sleep monitoring yields useful insights, it does not provide the same level of detail as specialized polysomnography assessments, which remain the gold standard in sleep research [40]. The noninvasive approach of consumer-grade devices, however, provides an accessible tool for identifying general trends in sleep pattern disruption that holds promise in health care research [18]. To gain a more comprehensive understanding of sleep quality, complementing wearable device research with information from gold standard clinical assessments is recommended [41]. Recognizing these limitations, future research using consumer-grade wearable devices for sleep monitoring may contribute to interventions aimed at optimizing sleep hygiene and could potentially lead to improved overall recovery outcomes.

### Study Limitations and Future Directions

While this pilot study demonstrates significant promise, several considerations and future directions merit attention. The relatively small sample size, the specific focus on adolescent student athletes, and the observational nature of this study limit the generalizability of the findings. A planned follow-up study will explore within- and between-group comparisons, as these were beyond the scope of this feasibility study. In addition, expanding the participant pool to include a broader age range and diverse athletic backgrounds would strengthen the external validity of study findings, as would matching cohorts by sport and including a healthy cohort of uninjured athletes. Additionally, longer-term investigations could shed light on the trajectory of recovery over extended periods and provide insights into the evolving role of consumer-grade wearable technology trends in the rehabilitation process. As noted earlier, the increasing use of consumer-grade wearable technology in athletes, the improving accuracy of devices over time, and the potential benefits of integration into sports medicine research prompt the need for studies to explore the utility of these devices in clinical research. Addressing issues such as data accuracy, participant adherence, and ethical considerations (eg, data security, privacy and confidentiality, and equity in device access) will be crucial in refining methodologies for future research endeavors.

### Conclusions

In conclusion, this study highlights that consumer-grade wearable technology is feasible for use in research with adolescent student athletes. These findings, along with their associated limitations, can inform the development of larger scale trials and, eventually, evidence-based protocols that can guide health care providers, coaches, and athletes in leveraging wearable technology to optimize injury recovery. The successful collection of physiological and biomechanical data through an easily accessible and consumer-friendly device lays the foundation for future investigations to explore trends in patient populations of interest with larger and more diverse cohorts. As wearable technology continues to advance and gain popularity, its integration into sports medicine research holds promise for improving the assessment and management of injured athletes.

## Abbreviations

HIPAA: Health Insurance Portability and Accountability Act
